# Cardiopulmonary Exercise Testing Is an Accurate Tool for the Diagnosis of Pulmonary Arterial Hypertension in Scleroderma Related Diseases

**DOI:** 10.3390/ph14040342

**Published:** 2021-04-08

**Authors:** Mattia Bellan, Ailia Giubertoni, Cristina Piccinino, Mariachiara Buffa, Debora Cromi, Daniele Sola, Roberta Pedrazzoli, Ileana Gagliardi, Elisa Calzaducca, Erika Zecca, Filippo Patrucco, Giuseppe Patti, Pier Paolo Sainaghi, Mario Pirisi

**Affiliations:** 1 Department of Translational Medicine, Università del Piemonte Orientale UPO, 28100 Novara, Italy; AiliaGiubertoni@hotmail.com (A.G.); mariachiarabuffa@gmail.com (M.B.); 20008635@studenti.uniupo.it (D.C.); ileanagagliardi91@gmail.com (I.G.); elisa.calzaducca91@gmail.com (E.C.); erikazecca24@yahoo.it (E.Z.); filippo_patrucco@hotmail.it (F.P.); giuseppe.patti@med.uniupo.it (G.P.); pierpaolo.sainaghi@med.uniupo.it (P.P.S.); mario.pirisi@med.uniupo.it (M.P.); 2 “AOU Maggiore della Carità” Hospital, 28100 Novara, Italy; cristina.piccinino@maggioreosp.novara.it (C.P.); daniele.sola@med.uniupo.it (D.S.); robepedra@gmail.com (R.P.); 3 CAAD, (Center for Translational Research on Autoimmune and Allergic Disease) Maggiore della Carità Hospital and Università del Piemonte Orientale UPO, 28100 Novara, Italy

**Keywords:** pulmonary arterial hypertension, systemic sclerosis, scleroderma, cardiopulmonary exercise testing

## Abstract

The early diagnosis of pulmonary arterial hypertension (PAH) is a major determinant of prognosis in patients affected by connective tissue diseases (CTDs) complicated by PAH. In the present paper we investigated the diagnostic accuracy of cardiopulmonary exercise testing (CPET) in this specific setting. We recorded clinical and laboratory data of 131 patients who underwent a CPET at a pulmonary hypertension clinic. Out of them, 112 (85.5%) had a diagnosis of CTDs; 8 (6.1%) received a diagnosis of CTDs-PAH and 11 (8.4%) were affected PH of different etiology. Among CPET parameters the following parameters showed the best diagnostic performance for PAH: peak volume of oxygen uptake (VO_2_; AUC: 0.845, CI95% 0.767–0.904), ratio between ventilation and volume of exhaled carbon dioxide (VE/VCO_2_ slope; AUC: 0.888, CI95%: 0.817–0.938) and end-tidal partial pressures (PetCO_2_; AUC: 0.792, CI95%: 0.709–0.861). These parameters were comparable among CTDs-PAH and PH of different etiology. The diagnostic performance was even improved by creating a composite score which included all the three parameters identified. In conclusion, CPET is a very promising tool for the stratification of risk of PAH among CTDs patients; the use of composite measures may improve diagnostic performance.

## 1. Introduction

Pulmonary arterial hypertension (PAH) is a progressive disease affecting the pre-capillary pulmonary vascular bed, leading to an increase in pulmonary vascular resistance and right ventricular failure, burdened by a high mortality rate [1]. PAH is a severe complication of different connective tissue disease (CTDs), particularly: systemic sclerosis (SSc), mixed connective tissue diseases (MCTD) and SSc overlapping with other CTDs [2]. The early diagnosis of SSc-PAH is difficult, since PAH is initially minimally symptomatic or asymptomatic, but absolutely pivotal: indeed, the early initiation of an effective treatment is the most relevant prognostic factor in patients affected by PAH [3]. This is why patients diagnosed with CTDs are commonly followed-up and screened for the development of PAH; the two-step algorithm DETECT is the most commonly used screening tool [4]. The DETECT algorithm includes a first step in which patients are indicated to echocardiography according to a composite score derived from the following variables: forced vital capacity (FVC) and diffusing capacity of the lung for carbon monoxide (DLCO); presence of teleangectasias and anti-centromere antibody; serum urate; N-terminal probrain natriuretic peptide and presence of right axis deviation on electrocardiogram. On the basis of the result of echocardiography, at risk patients will be further tested with right heart catheterization (RHC). The detect score well performs in this setting, showing a very high sensitivity (96%), as required by any screening tool; however, the specificity is low (48%) having as a direct consequence the need for a high number of unnecessary invasive measurement of pulmonary pressure, by RHC [5]. This is why there is an unmet need of novel biomarkers able to refine the PAH risk stratification among CTDs patients [6,7]. In the last years, cardiopulmonary exercise testing (CPET) has been proposed as a novel tool to better select those patients at higher risk of PAH, thus requiring RHC [8]. CPET provides an important insight into exercise physiology and, according to recent data, may contribute to the identification of PAH among SSc patients [9]. In the present study we aimed to confirm this observation and to evaluate whether CPET findings among PAH-CTDs patients differ from patients affected by PH of a different etiology, in a pilot study.

## 2. Results

We recruited 131 patients, 115 (87.8%) females; the median age was 61.5 (52.0–69.5) years. Out of them, 112 (85.5%) had a diagnosis of CTDs alone: 84 were affected by SSc, 15 by overlap syndrome, 5 by MCTD and 8 by Undifferentiated connective tissue disease (UCTD). The median disease duration of CTDs was 5 (2–11) years; antirheumatic treatment mainly included hydroxychloroquine (*N* = 57, 50.9%) and methotrexate (*N* = 15, 13.4%). 37 patients (33.0%) were receiving steroids when CPT was performed.

8 (6.1%) received a diagnosis of CTDs-PAH: 6 were affected by SSc, 1 by overlap syndrome and 1 by MCTD; 6 patients were on endothelin receptor antagonists, 5 patients were receiving phosphodiesterare 5 inhibitors. Moreover 1 patient was receiving riociguat and 1 patient selexipag. The median time to pulmonary hypertension diagnosis was 5 (3–6) years.

Finally, 11 (8.4%) patients received a diagnosis of PH of different etiology (5 PAH, 4 chronic thromboembolism, 1 unknown).

**Figure 1:**
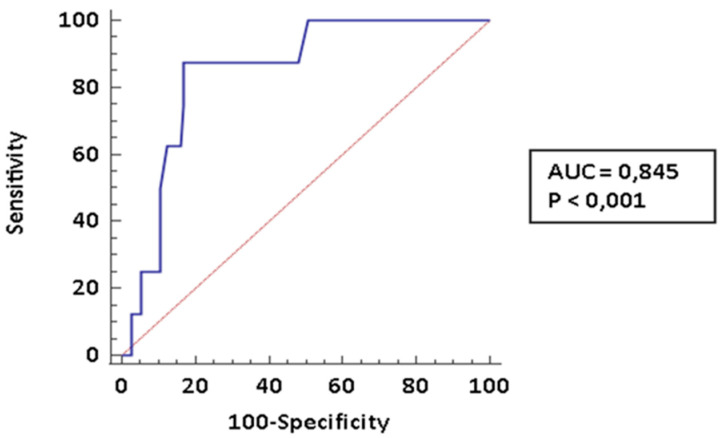
ROC curve for peak VO_2._ We diagnosed 8 CTD-PAH.

In Table 1 we report the main clinical and laboratory features of the three study groups:

As shown in the Table 1, patients affected by PH show higher sPAP and lower DLCO, as expected. Among CTD patients, those with PAH are significantly older. Looking at the CPET parameters, we compared the results of the test among groups. The results are shown in Table 2:

Patients with PH have a significantly lower peak VO_2_ and basal PetCO_2_, and a significantly higher VE/VCO_2_ slope. These parameters were comparable among CTDs-PAH patients and PH patients with different etiologies. We finally evaluated the diagnostic accuracy of the different CPET parameters considered. In Figure 1 we reported the ROC curve for peak VO_2_ for the diagnosis of PAH in patients affected by CTDs. As shown, this parameter has very good diagnostic performance (AUC: 0.845; CI 0.767–0.904); a value ≤ 14.1 is 87.5% sensitive (LR− 0.15, CI95% 0.02–0.90) and 83.05% specific (LR+ 5.16, CI95%: 3.2–8.4) for PAH. The NPV is 98.4% (CI 95% 90.5–99.7%), while the PPV is 36.4% (CI 95% 26.1–48.2%).

**Figure 2:**
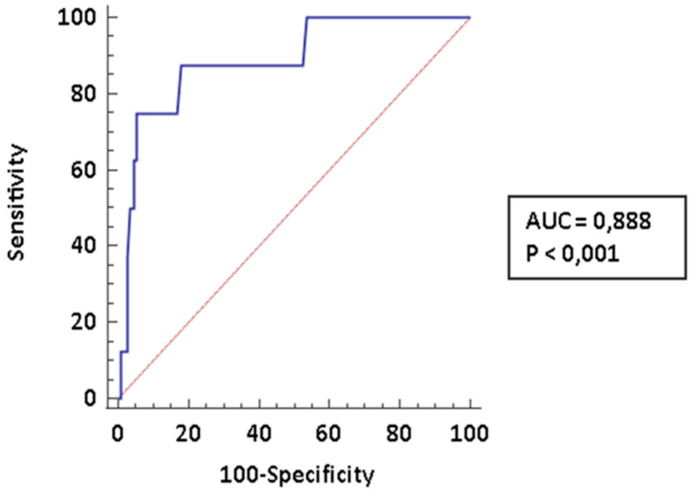
ROC curve for peak VE/VCO_2_ slope. We diagnosed 8 CTD-PAH. For abbreviation: AUC, Area under the curve.

In Figure 2 we reported the ROC curve for VE/VCO2 slope, which again demonstrates a very good diagnostic accuracy (AUC: 0.888; CI95%: 0.817–0.938); a threshold > 33.96 is 87.5% sensitive (LR− 0.15, CI95% 0.02–1.0) and 82.14% specific (LR+ 4.9, CI95%: 3.0–7.9) specific for PAH. The NPV is 98.3% (CI95% 90.4–99.7) and the PPV is 35.3 (CI95%: 25.3–46.7).

**Figure 3:**
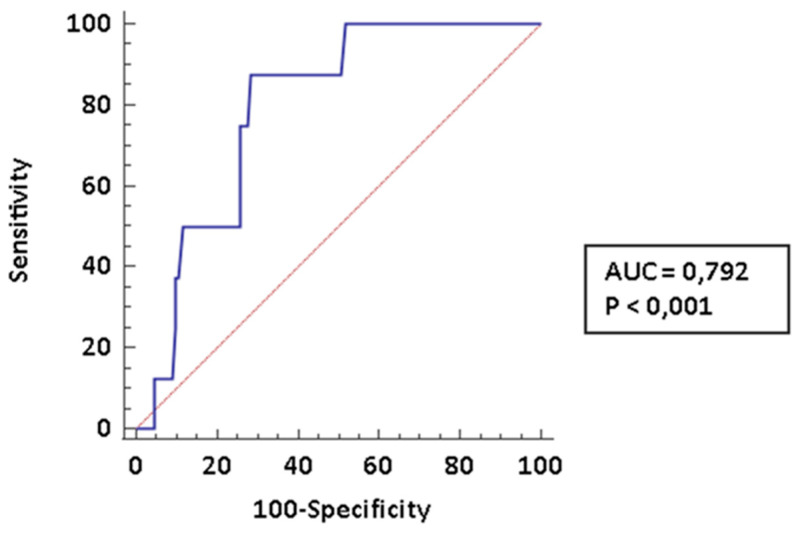
ROC curve for basal PetCO_2._ We diagnosed 8 CTD-PAH.

Moreover, in Figure 3 we reported the ROC curve for basal PetCO2; a threshold ≤ 27.2 is 87.5% sensitive (LR− 0.18; CI95% 0.03–1.1) and 71.43% specific (LR+ 3.1; CI95%: 2.1–4.5) specific for PAH, while the AUC is: 0.792 (CI95%: 0.709–0.861). The PPV is 25.4% (CI 95% 18.7–33.5) and the NPV is 98.1% (89.1–99.7).

We finally tried to evaluate the diagnostic accuracy of a composite predictive model including all these three parameters. We scored 1 point for each parameter considered: a peak VO_2_ ≤ 14.1; a VE/VCO_2_ slope > 33.96; a basal PetCO_2_ ≤ 27.2. In Table 3 we report the different scores according to the presence/absence of PAH among CTDs patients. The distribution was significantly different (χ^2^ for trend; 34.3 *p* < 0.0001).

**Figure 4:**
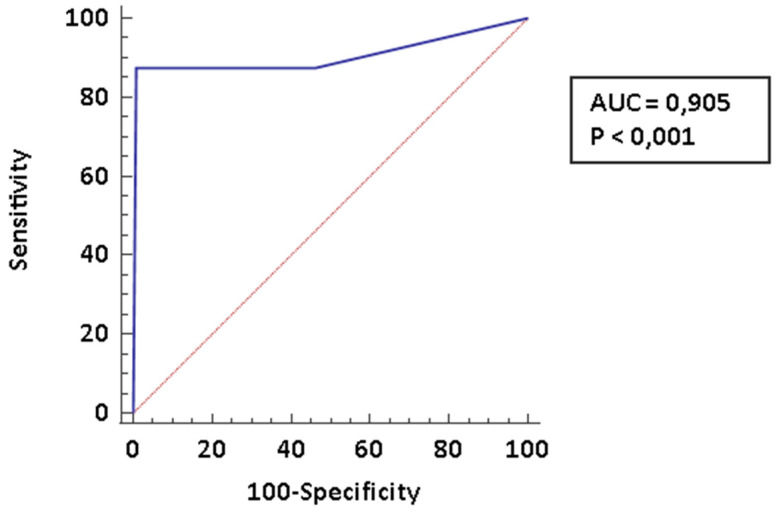
ROC curve for the composite model. We diagnosed 8 CTD-PAH.

As shown in the ROC curve (Figure 4), a score of 3 is 87.5% (CI95% 47.3–99.7) sensitive and 99.1% (CI95%:95.1–100.0) specific for PAH. The LR− is 0.13 (CI95%: 0.02–0.8) and the LR+ 98 (CI 95%: 13.7–701.8). The PPV is 91.6% (CI 95%: 60.3–98.7) and the NPV is 98.6% (CI 95%: 91.9–99.8).

## 3. Discussion

In the present paper we aimed at preliminary evaluating the diagnostic accuracy of CPET in the diagnosis of PAH among CTDs patients. According to our data, CPET is a potentially sensitive and specific tool for the diagnosis of pulmonary hypertension, independently from the underlying etiology. The use of composite, CPET-based scores might improve the diagnostic accuracy and should be evaluated on a larger scale, since our pilot study provides only preliminary data. These findings will be herein discussed on the basis of the current literature.

SSc is a potentially severe condition; according to a meta-analysis published in 2012, which pooled data from different cohort studies covering over 50 years of observation, patients affected by SSc have a standardized mortality ratio 3.5 times higher than the general population [10]. PAH is a major determinant of this excess of deaths; in fact, according to the EUSTAR cohort study, more than half SSc patients dies because of a condition related to their underlying CTD; more specifically, around 15% of deaths are related to the development of PAH [11]. Patients affected by PAH have a severe prognosis; the annual mortality is around 10% in idiopathic PAH [3] and even worse in CTDs-related PAH, particularly when SSc is the underlying rheumatic condition [12]. Indeed, according to Mukerjee et al., the survival rate at 1-, 2- and 3-years was respectively 81%, 63%, and 56% [13] and a comparable prognosis has been reported more recently in different registry based studies [14,15]. 

The degree of hemodynamic impairment is universally considered a main prognostic predictor in PAH [3,14,15,16]; being available different therapeutic strategies effective in the management of PAH, it is reasonable to consider early diagnosis fundamental to impact on patients’ prognosis. This is why SSc patients should undergo a regular screening strategy to early identify cardiopulmonary involvement and to start the treatment as soon as possible. According to the lastly updated Eular guidelines for the management of SSc-PAH, the treatment of this condition should include the same classes of drugs used in the other forms of PAH; this recommendation belongs to the results of different high quality clinical trials including heterogeneous population of PAH patients among whom CTDs-PAH was included [17]. 

The most commonly used screening protocol is the DETECT algorithm, firstly described by Coghlan et al. in 2014; it is a very well performing diagnostic tool, particularly in the context of a screening strategy, because of its optimal sensitivity. However, it should be acknowledged that its specificity is quite low. This is not negligible; in fact, the relatively low positive predictive value accounts for an excess of patients needing to be tested with the gold standard for PAH diagnosis, the RHC. This test is invasive and burdened by potential complications which, although rare, may be severe and even fatal [18]; thus, a better tailoring of screening strategy and individual’s risk stratification may allow to reduce the costs and limit the risk for patients, by reducing the number of patients unnecessary tested.

In the last few years an increasing number of papers investigated the diagnostic role of CPET in the identification of those CTDs patients with PAH [19]. Although this test is not currently included in the guidelines for the management of SSc, the current literature is highly consistent in confirming its potential in the identification of PAH in this specific group of individuals. 

In our study, we selected the best performing parameters and finally we built a composite score to improve their diagnostic accuracy. We reported that peak VO_2_ is significantly reduced in CTDs-PAH, with respect to CTDs alone; this reduction is comparable to what observed in patients affected by PH with different etiologies. Similar results were previously reported by Dumitrescu et al. and may be explained by the fact that peak VO_2_ is closely related to cardiac output during exercise; therefore, a high peak VO_2_ reflects a good hemodynamic adaptation to exercise and is able to rule out PAH with high accuracy. The authors also evaluated the best performing threshold for this parameter, identifying a cut-off of 13.8, very close to our one [8]. Another common finding is the good clinical performance of VE/VCO_2_ slope. In a recent paper of an Italian group, VE/VCO_2_ slope was the best parameter able to identify PAH at RHC, on top of a positive DETECT screening. Interestingly, once more, the cut-off used was quite close to the one that we identified (35.5 vs. 33.9) and showed an optimal sensitivity and a good specificity yielding a PPV of 0.636 (0.556–0.750) [9]. We also tested the role of PetCO_2_; as already reported by Dumitrescu et al. [8], this marker of ventilator efficiency is predictive of PAH, although its diagnostic power is lower than peakVO_2_. Interestingly, these alterations are not specific for CTDs-PAH; we, indeed, included a subgroup of subjects suffering for PH of different etiologies, demonstrating that the CPET is more generally able to detect cardiopulmonary involvement. A major novel finding of our work is the observation that combining different CPET parameters may improve the diagnostic accuracy of the test; in particular, we propose a 3-points score based on the three previously discussed parameters which very well fits to our population. Despite having a sensitivity which is similar to the one of any single parameter tested, the use of a composite score significantly enhances the specificity and the PPV. This may, finally, significantly increase the diagnostic accuracy of the present tool. On this basis, we can postulate that CPET may contribute to a better stratification of those patients really requiring a RHC; it can be argued that, in the context of a screening procedure, the use of less sensitive tools than DETECT algorithm can cause the loss of some PAH cases with relevant clinical implications; however, RHC is an invasive procedure. Thus, we might hypothesize that RHC may be postponed in those patients with an indication according to DETECT but with a normal CPET. These subjects may be addressed to a stricter follow-up to early identify clinical deterioration. Moreover, we can also postulate that, giving the very high PPV of CPET, it could even represent an alternative to RHC in those patients at higher procedural risk. However, we acknowledge that the low number of cases and the cross-sectional design of the study limit the possibility to truly test our score as a diagnostic tool. Prospective studies are indeed required to evaluate its potential clinical application.

A further element of discussion belongs to the observation that SSc patients may have less marked alteration of CPET even in absence of PAH; according to previous findings, in fact, patients with SSc may show increased VE/VCO_2_ slope and decreased peak VO_2_ with respect to the general population [20]. It will be interesting to evaluate, in the next future, whether those patients with an altered CPET may represent a subset of individuals at higher risk for PAH development. Obviously, our study because of its cross-sectional design is not able to give an answer to this relevant research question. 

Our paper has some limitations: first of all, the number of patients affected by PAH included in the final analysis is low; our study should be, in fact, considered for what it actually is: a pilot study with preliminary results that, although promising, require a confirmation on a larger scale. Furthermore, some of the patients received a diagnosis of pulmonary hypertension on the basis of the echocardiographic findings, being RHC contraindicated. Despite this is in line with the current international guidelines, we should acknowledge that RHC is the gold standard for the diagnosis of pulmonary hypertension. Moreover, the diagnostic efficacy of this CPET-based score should be tested in a more comprehensive strategy, as integration of the standard DETECT algorithm. A further relevant limitation is that we considered prevalent PAH, rather than incident PAH; patients were not naïve to treatment, which might have affected our findings. 

## 4. Materials and Methods

We performed a cross-sectional, observational study on patients evaluated at the Pulmonary Hypertension Clinic of the Cardiology Division, University Hospital of Novara from 3 October 2016 to 12 December 2019. The clinic was the main referral for Rheumatology Units of the geographic area, representing a major facility for PAH screening of CTDs patients. The study protocol was approved by the local ethical committee and conducted in strict accordance with the principles of the Declaration of Helsinki. Written informed consent was obtained from all individual participants included in the study.

We included all the patients older than 18 years who underwent, under clinical indication a CPET. We excluded from the study those who refused to sign the informed consent. We included in the study both CTDs patients and patients with a diagnosis of PH with a different etiology. The following criteria were applied to classify the different rheumatic conditions:

- SSc: 2013 ACR/Eular classification criteria [21];

- MCTD: Kasukawa’s criteria [22];

- Overlap syndrome: patients fulfilling the classification criteria for SSc along with those of other rheumatic conditions [23];

- UCTD was made when patients with a connective tissue disease did not meet the classification criteria of any specific syndrome [24]. 

All the included patients also underwent a comprehensive clinical evaluation and a biochemistry panel; moreover, respiratory function and echocardiography were performed as described in previous papers belonging to the same project [6,7].

We recorded the following standardized measurements: forced vital capacity (FVC), forced expiratory volume in one second (FEV1), and FEV1/FVC%), diffusing capacity of the lung for carbon monoxide adjusted for alveolar volume (DLCO VA), measured with the single-breath Jones-Meade protocol, corrected for alveolar ventilation, systolic pulmonary pressure (sPAP), right atrium area (RAA), right ventricle diameter (RVD), and ejection fraction (EF).

According to the application of international guidelines, those patients with a suspected PAH underwent right heart catherization. PAH was defined by mean pulmonary artery pressure (mPAP) ≥ 25 mmHg, pulmonary capillary wedge pressure ≤ 15 mmHg, and pulmonary vascular resistance >3 wood units. Whenever contraindications to RHC occurred, pulmonary hypertension was diagnosed based on echocardiography-estimated sPAP ≥ 35 mmHg and additional high probability criteria (1 patient in the group B and 5 patients in the group C), in agreement with the 2015 ESC/ESR guidelines [1].

CPET was performed on a stationary bicycle ergometer, within two weeks from echocardiographic assessment and PFTs. The exercise protocol consists of 3 min of rest followed by the incremental work rate to the patients’ maximum tolerance, then 5 min of recovery. The incremental work rate was selected according to the patient’s exercise capacity to aim for 8–12 min in length. Gas exchange was measured breath-by-breath during the test using a Schiller Cardiovit CS-200 Ergo-Spiro System (Baar, Switzerland); we used the Ganshorn Medizine Eletronic software for pulmonary function testing (v. LF8.5M SR3, Niederlauer, Germany). Equipment was calibrated before each exam. ECG and pulse oximetry were continuously monitored, and blood pressure was measured every three minutes. Minute ventilation (VE), heart rate (HR), oxygen uptake (VO_2_), carbon dioxide production (VCO_2_), CO_2_ ventilatory equivalent (VE/VCO_2_ or EQCO_2_), O_2_ ventilatory equivalent (VE/VO_2_ or EQO_2_), end tidal O_2_ (PetO_2_), end tidal CO_2_ (PetCO_2_), tidal volume and PulseO_2_ were averaged every 10 s. Predicted value for peak VO_2_ were calculated according to the standard formula. The first ventilatory threshold was determined from gas exchange by the V-slope method, derived from the plot with VO_2_ and VCO_2_ recognizing the point where VCO_2_ started increasing faster than VO_2_, in all patients. The relationship between VE and VCO2 (VE/VCO_2_ slope) was calculated as the slope of the linear relationship between VE and VCO_2_ from one minute after the beginning of loaded exercise to the end of the isocapnic buffering period. We considered maximal effort ad achieved if the respiratory exchange ratio (RER) calculated as the ratio between VO_2_ and VCO_2_ was above 1,10. All CPET were executed and analyzed by one physician’s blinded to patients’ clinical features.

### Statistical Analysis

All the data were recorded in a database and analyzed by the statistical software package MedCalc v.19.6.4 (MedCalc Software, Broekstraat 52, 9030, Mariakerke, Belgium). Continuous variables are presented as medians and interquartile range [IQR]. We compared continuous variables among groups by Kruskal-Wallis test, while categorical distribution was tested by Pearson’s χ^2^.

To test the diagnostic performance of different CPET parameters among CTDs patients, receiver operating characteristics curves were built, with calculation of the areas under the curve (AUC). Moreover, we calculated the sensitivity, specificity, positive and negative likelihood ratio (LR+ and LR−), negative predictive value (NPV) and positive predictive value (PPV) for the different thresholds. NPV and PPV were calculated on the basis of an estimated rate of CTDs-PAH of 10%. The best diagnostic thresholds were identified according to the Youden index J and used to build a composite score which was tested for its diagnostic performance.

The level of significance chosen for all statistical analysis was 0.05 (two-tailed).

## 5. Conclusions

In conclusion, our paper supports the idea that CPET should be considered for the extensive use in the follow-up of CTDs patients at risk for PAH; a multiparametric diagnostic strategy might be more effective to improve the diagnostic performance of this examination.

## Figures and Tables

**Table 1:** Main general features of the study population and comparison between groups. For abbreviation: CTDs, connective tissue diseases; PAH, pulmonary arterial hypertension; PH, pulmonary hypertension; M, males; F, females; FVC, forced vital capacity; FEV1, forced expiratory volume in the 1st second; DLCO, diffusing capacity of the lung for carbon monoxide; LVEF, left ventricular ejection fraction; sPAP, systolic pulmonary arterial pressure. *A vs. B; ^§^ A vs. B and C.

	Group A CTDs	Group B CTDs-PAH	Group C PH	*p*
**Gender, M/F**	11/101	1/7	4/7	0.04
**Age, years**	61 (50–68)	70.5 (68–73.5)	65 (56–78)	0.01 *
**FVC, % of predicted value**	104 (92–116)	99 (89–126)	87 (67–106)	0.21
**FEV1, % of predicted value**	104 (91–115)	117 (90–124)	87 (74–111)	0.16
**DLCO, % of predicted value**	89 (77–96)	48 (46–62)	76 (64–79)	<0.0001 ^§^
**LVEF, %**	63 (59–66)	64 (63–66)	57 (54–65)	0.18
**sPAP, mmHg**	26 (23–30)	46 (38–65)	47 (39–53)	<0.0001 ^§^

**Table 2:** Comparison of the main CPET parameters among groups between groups. For abbreviation: VO_2_, volume of oxygen uptake; VE, ventilation; VCO_2_, volume of exhaled carbon dioxide; PetCO_2_, end-tidal partial pressures for CO_2_. *A vs. B; ^§^ A vs. B and C; ° C vs. A and B.

	Group A CTDs	Group B CTDs-PAH	Group C PH	*p*
**Peak VO2 (ml/kg/min)**	18.4 (15.1–21.8)	12.5 (12.0–14.0)	11.6 (9.2–17.0)	<0.0001 ^§^
**VO2 at first ventilator threshold (% of peak VO2)**	56 (49–64)	55 (48–58)	64 (24–73)	0.25
**VE/VCO2 slope**	29.1 (26.4–32.6)	40.4 (36.3–41.2)	37.1 (31.6–51.6)	<0.0001 ^§^
**PetCO2 basal (mmHg)**	29.2 (26.1–31.0)	25.0 (23.2–26.9)	25.9 (22.9–27.6)	0.005 ^§^
**Pulse O2 peak (%)**	82 (73–92)	66 (63–75)	89 (52–92)	0.04 *
**EQCO2 basal**	37 (34–42)	42 (38–45)	41 (38–48)	0.04
**Duration of exercise, minutes**	10 (8.5–12)	7 (4.5–9.5)	8 (6.5–10)	0.004 ^§^
**Maximal workload, watts**	68.5 (52.0–89.0)	37.0 (32.5–50.0)	56.0 (35.5–88.0)	0.02 *
**Respiratory exchange ratio**	1.0 (1.0–1.0)	1.0 (0.5–1.0)	1.1 (1.0–1.2)	0.004 °

**Table 3:** Application of the CPET scoring system among CTDs patients.

	Score = 0	Score = 1	Score = 2	Score = 3
CTDs	60 (53.6%)	34 (30.4%)	17 (15.2%)	1 (0.9%)
CTDs-PAH	1 (12.5%)	0	0	7 (87.5%)

## Data Availability

Data are available upon reasonable request to the corresponding author.
